# ΔR1_blood_, a surrogate of blood-pool gadolinium concentration, is related to body mass index, gender, left ventricular end-diastolic volume index, cardiac index, and field strength at cardiac magnetic resonance late enhancement imaging

**DOI:** 10.1016/j.jocmr.2025.101929

**Published:** 2025-06-25

**Authors:** Patrick Doeblin, Shing Ching, Wensu Chen, Natalia Solowjowa, Stefanie Maria Werhahn, Rebecca Elisabeth Beyer, Misael Estepa, Christian Stehning, Jeffrey Ji-Peng Li, Henryk Dreger, Sebastian Kelle

**Affiliations:** a Department of Cardiology, Angiology and Intensive Care Medicine, Deutsches Herzzentrum der Charité, Augustenburger Platz 1, 13353 Berlin, Germany; b Charité – Universitätsmedizin Berlin, corporate member of Freie Universität Berlin and Humboldt-Universität zu Berlin, Charitéplatz 1, 10117 Berlin, Germany; c DZHK (German Centre for Cardiovascular Research), partner site Berlin, Potsdamer Str. 58, 10785 Berlin, Germany; d Division of Cardiology, Department of Medicine and Geriatrics, United Christian Hospital, Hong Kong, China; e Department of Cardiology, Affiliated Hospital of Xuzhou Medical University, Xuzhou, China; f Department of Cardiothoracic and Vascular Surgery, German Heart Center Berlin, Berlin, Germany; g Clinical Science, Philips Healthcare, Röntgenstraße 24, 22335 Hamburg, Germany

**Keywords:** Gadolinium, Gender, BMI, T1 Mapping

## Abstract

**Background:**

Late gadolinium enhancement imaging is the cornerstone of tissue characterization via cardiac magnetic resonance imaging. The contrast-enhancing effect of gadolinium is caused by a linear increase in tissue longitudinal R1 relaxation rates (R1 = 1/T1). The change in R1 of blood pre- and post-contrast (ΔR1_blood_) is therefore a surrogate for the blood-pool gadolinium concentration, which in turn correlates linearly to the tissue gadolinium concentration. The total volume of distribution for gadolinium is the extracellular volume of the body, which differs with body composition, potentially leading to variations in blood-pool and tissue gadolinium concentrations.

**Methods:**

This study is a hypothesis-generating secondary analysis of a dataset of 1098 patients who underwent contrast cardiovascular magnetic resonance between August 2014 and November 2020 at a tertiary center. ΔR1_blood_ was calculated from T1 relaxation time maps acquired before and approximately 15 min after application of 0.15 mmol/kg gadobutrol. Explorative data analysis and multiple linear regression were performed to assess the influence of body mass index (BMI), gender, age, cardiac index (CI), hematocrit (Hct), and left ventricular end-diastolic volume index (LVEDVi) on ΔR1_blood_.

**Results:**

In bivariate analysis, ΔR1_blood_ showed moderate correlation to BMI and weak correlation to LVEDVi, Hct, and CI. The correlation to BMI was higher in women (r = 0.52 at 1.5T and r = 0.47 at 3T) than in men (r = 0.27 at 1.5T and r = 0.37 at 3T). Multiple linear regression showed independent predictive value of BMI, BMI:gender, gender, CI, field strength (FS), and LVEDVi (R² = 0.268, *P* < 0.001), with BMI remaining the strongest individual predictor (b = 0.032 [0.025; 0.040], η² = 0.13, *P* < 0.001).

**Conclusion:**

ΔR1_blood_, a measurement of gadolinium contrast enhancement in the blood-pool and a surrogate of plasma C_Gd_ at the time of late enhancement imaging, showed moderate association with BMI, FS, and gender and weak association with LVEDVi and CI. Further research is necessary to assess the need for individualized gadolinium dosing.

## Graphical abstract

**Figure:**
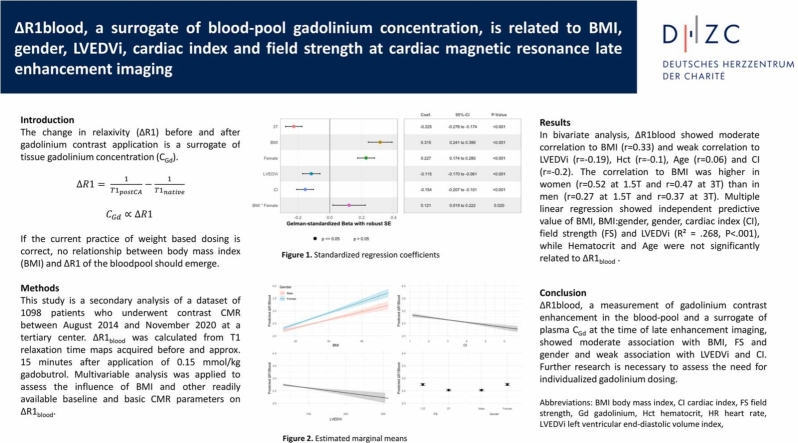

## 1 Background

Late gadolinium enhancement (LGE) imaging is a cornerstone of cardiovascular magnetic resonance (CMR) tissue characterization. It enables the detection and differentiation of both ischemic and non-ischemic myocardial injury, carries strong prognostic value, and guides therapy in a variety of cardiac diseases [1]. LGE imaging requires the application of an extracellular gadolinium-based contrast agent, as image contrast is based on local tissue differences in extracellular volume (ECV). At the time of LGE imaging, the contrast agent concentration (C_Gd_) has reached an equilibrium between the intravasal and extravasal ECV. While the C_Gd_ within the ECV is equal throughout the body, tissue C_Gd_ differs according to tissue ECV, with higher gadolinium concentrations in tissues with higher ECV, such as scar, fibrosis, and necrosis. This higher tissue C_Gd_ translates to higher signal on LGE imaging, allowing differentiation of fibrotic or necrotic tissue from healthy myocardium.

The recommended contrast agent dose for LGE imaging is 0.1 to 0.2 mmol of an extracellular gadolinium-based contrast agent per kg of patient body weight [1]. This weight-based dosing recommendation relies on the presumption that the total ECV of the body is linearly correlated to the total body weight. But while total body weight is a large determinant of total ECV, the latter might also be influenced by other variables. In a study of 1818 healthy volunteers, the mean total ECV in percentage of total body weight was 24.2% for lean men and 20.0% for lean women, and 23.4% for obese men and 18.6% for obese women, highlighting potentially relevant differences in ECV based on gender and body composition [2]. Older age has been related to decreased total body water, although the effect on extracellular water is less clear [3], [4]. Differences in total ECV could translate to variations in blood-pool C_Gd_. Other factors potentially influencing the blood-pool C_Gd_ at the time of LGE imaging are the hematocrit (Hct), the rate of distribution, the rate of elimination, the contrast dose, and the time elapsed from contrast application to LGE imaging. Cardiac output (CO) has been shown to affect the pharmacokinetics of narcotics, but research on C_Gd_ is lacking [5].

Variations in blood-pool C_Gd_ could be clinically relevant for several reasons. First, underdosing affects image quality, leading to a reduced signal-to-noise ratio (SNR) [6] [7]. This could cause underestimation of scar size both visually and via SNR-based thresholds. Second, estimates of myocardial ECV by T1 mapping are influenced by the contrast dose and therefore susceptible to C_Gd_ [8]. Third, the risk of nephrogenic systemic fibrosis in patients with renal failure increases with the gadolinium dose, so that the lowest possible dose to achieve the desired contrast-enhancing effect should be applied [9]. We therefore examined the change in blood-pool T1 relaxation rate (ΔR1_blood_), which is directly proportional to and therefore a surrogate of blood-pool C_Gd_, in relation to gender, age, body mass index (BMI), Hct, left ventricular end-diastolic volume index (LVEDVi), and cardiac index (CI).

## 2 Methods

### 2.1 Patients

This is a secondary analysis of a retrospective study of patients who underwent CMR between August 2014 and November 2020 at our institution and had pre- and post-contrast T1 mapping and Hct measurements performed within 24 h of scanning. The primary analysis consisted of 652 patients examined at a clinical 3T MRI scanner (Ingenia, Philips Healthcare, Best, The Netherlands) and 449 patients examined at a 1.5T MRI scanner (Achieva, Philips Healthcare, Best, The Netherlands). For 22 patients with missing height or weight data in the original dataset, the respective values were retrieved from the patient records. For three patients, no weight or height data were available, leaving a total of 1098 patients for the current analysis.

### 2.2 CMR acquisition

All examinations were performed for various clinical indications on abovementioned MRI scanners. Details of the CMR protocol have been described previously [10]. Briefly, cine images were acquired using retrospectively gated cine-CMR in cardiac short-axis, vertical long-axis, and horizontal long-axis orientations using a balanced steady-state free precession sequence. T1 mapping using the MOdified Look–Locker Inversion-recovery (MOLLI) sequence with a 5s(3s)3s—scheme was performed before and approximately 15 min after application of 0.15 mmol/kg gadobutrol (Gadovist®, Bayer AG, Leverkusen, Germany) [11]. The contrast dosing scheme, but not the total dose, varied depending on the indication. For stress perfusion imaging, a bolus of 0.0375 mmol/kg was used at 3T and 0.075 at 1.5T per perfusion. Rest perfusion was performed only upon request of the physician. After the perfusion scans were completed, any remaining gadobutrol to achieve a total dose of 0.15 mmol/kg was administered as a single bolus. For all other indications, a single bolus of 0.15 mmol/kg gadobutrol was administered. Regardless of the dosing scheme applied, late enhancement imaging commenced 10 min after the last contrast bolus, followed by post contrast T1 mapping.

### 2.3 CMR analysis

The analysis of the MOLLI sequences for T1 relaxation times and cine images for left ventricular volumetric measurements was performed using commercially available postprocessing software (Intellispace Portal Version 11.1, Philips Healthcare, Best, The Netherlands) as described previously [10]. Indexed values were calculated by dividing the unindexed value by the body surface area (BSA). CO was calculated as the product of left ventricular stroke volume and heart rate.

### 2.4 Measurement of ΔR1_blood_

ΔR1_blood_ was calculated from left ventricular blood-pool pre- and post-contrast T1 relaxation times as follows:

$$∆R1[s^{-1}]=\;\frac{1000}{{T1}_{\mathrm{post CA}}[\mathrm{ms}]}-\;\frac{1000}{{T1}_{\mathrm{native}}[\mathrm{ms}]}$$

### 2.5 Selection of predictor variables

**Fig. 1:**
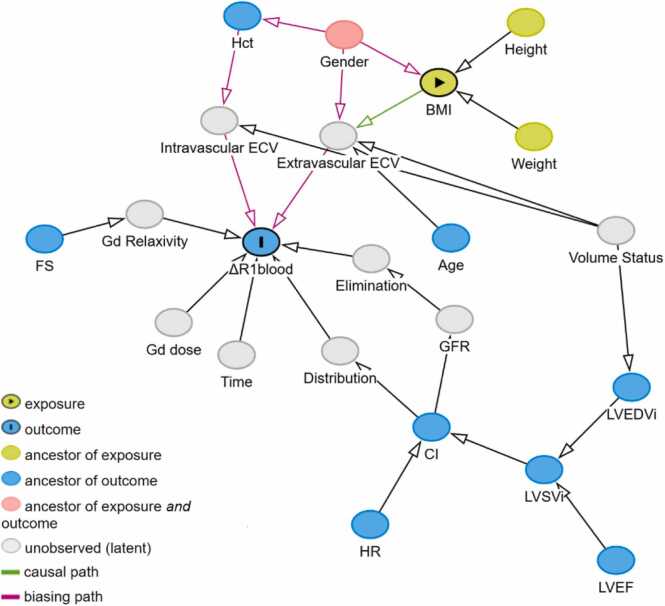
Directed acyclic graph for the influence of BMI and possible confounders on ΔR1_blood_. BMI was defined as exposure, ΔR1_blood_ as outcome. Ancestors of exposure are variables that (directly or indirectly) influence BMI but not ΔR1_blood_ (height and weight). Ancestors of outcome are those variables that influence ΔR1_blood_ but not BMI (Hct, FS, age, CI, HR, LVSVi, LVEF, LVEDVi). Confounders are variables that influence both BMI and ΔR1_blood_ (gender) and must be controlled for. Unobserved variables are those for which no data were available (extra-/intracellular ECV, Gd relaxivity, elimination, distribution, time, Gd dose) or which were constant for all patients (Gd dose). No unobserved confounders were identified. Mediators are variables that lie between exposure/confounder and outcome (Gd relaxivity, extra-/intravascular ECV, Gd relaxivity, elimination, distribution). Correcting for mediators is not necessary to assess the relation between exposure and outcome. The resulting model must therefore at minimum include all exposure and confounder variables. *BMI* body mass index, *CI* cardiac index, *ECV* extracellular volume, *FS* field strength, *Gd* gadolinium, *GFR* glomerular filtration rate, *Hct* hematocrit, *HR* heart rate, *LVEDVi* left ventricular end-diastolic volume index, *LVEF* left ventricular ejection fraction, *LVSVi* left ventricular stroke volume index

Based on a-priori knowledge of physiological mechanisms and a directed acyclic graph (Fig. 1), the influence of the following variables on ΔR1_blood_ was examined:

- BMI and gender, due to the presumed differences in extra- and intravascular ECV. The term BMI:gender was included because changes in BMI are presumed to affect body composition differently in men than in women.
- Field strength (FS) to control for differences in contrast agent relaxivity.
- CI to assess whether CO affects gadolinium distribution at the time of late enhancement imaging. Indexed values were used due to the known dependency of the CO on BSA.
- LVEDVi to assess whether volume overload affects ΔR1_blood_ due to an increase in extracellular water.
- Age to assess whether age-related differences in body composition affect ΔR1_blood_.
- Hct to assess whether ΔR1_blood_ is related to differences in plasma volume.

### 2.6 Statistical analysis

Continuous variables were expressed as mean and standard deviation, categorical variables as sum and percentages. The directed acyclical graph was created using DAGitty (Version 3.1, Radboud University, Nijmegen, The Netherlands) [12].

Descriptive data analysis for ΔR1_blood_ and all a-priori defined predictor variables included both uni- and bivariate analysis. Univariate analysis was performed with histograms and QQ plots. Bivariate analysis was performed using Pearson product-moment correlation analysis for the combination of two continuous variables, boxplots for the combination of a continuous variable with a factorial variable, and mosaic plots for the combination of two factorial variables. Additionally, the bivariate relationship of ΔR1_blood_ and BMI was assessed in groups separated by gender and FS to assess interaction. For bivariate correlations, categories adapted from Cohen were used to describe the effect size of the correlation coefficient r: ∼0.10 weak, ∼0.30 moderate, and ∼0.50 large [13].

We conducted a linear regression analysis to examine the effects of all a-priori defined factors and centered covariates on ΔR1_blood_, conceptually equivalent to analysis of covariance. Centering was performed before building interaction terms. The model was further refined by recursive feature elimination based on statistical significance (cutoff: P > 0.05). For interaction terms, the constituent main effects were retained in the model regardless of their statistical significance. Effect sizes of the final model were assessed using partial eta squared (ηp²) as a measure of explained variance by each individual predictor, with the following categories according to Cohen: ∼0.01 small, ∼0.06 medium, and ∼0.14 large [14].

The residuals of the final model were tested for normality and homoskedasticity, both visually using a histogram, QQ plot, and scatter plot of actual vs predicted residuals as well as a Shapiro-Wilks and a Breusch-Pagan test. Deviations from the normal curve in input variables as well as the residuals of our final model were tolerated, as the test statistic can be considered approximately normally distributed due to the large sample size in conjunction with the central limit theorem. Because residual plots indicated slight heteroskedasticity, we computed heteroskedasticity-consistent robust standard errors (SE) using the HC3 estimator via the sandwich package in R [15]. A sensitivity analysis was performed by trimming outliers based on a Cook-distance of 4/n.

Both unstandardized and standardized parameter estimates were reported. Continuous predictors were standardized by subtracting the mean and dividing by 2 standard deviations, following Gelman, to facilitate comparison with binary predictors [16]. A fully standardized model, using 1 standard deviation and standardized outcome, is given in the Supplementary Material. All standardization was performed before creating interaction terms.

Centered values were back-transformed for a regression equation with raw values. Estimated marginal means and effects were reported for the final model, with 95% confidence bands based on robust SE (HC3).

The primary endpoint was the significance of the final (unstandardized) model. Two-tailed tests were used where appropriate, with a significance level of α = 0.05. No corrections for multiple testing were performed. The data were analyzed using R version 4.5.0 (The R Foundation for Statistical Computing, Vienna, Austria).

## 3 Results

### 3.1 Baseline characteristics

The baseline characteristics of the 1098 patients are shown in Table 1. BMI was numerically higher in men than in women (26.9 ± 4.5 vs 25.3 ± 5.4 kg/m²) and at 3T compared to 1.5T (26.9 ± 5.0 vs 25.5 ± 4.6 kg/m²) due to the larger bore of the 3T scanner, allowing for the examination of larger patients. ΔR1_blood_ was numerically lower in men than in women (2.50 ± 0.45 vs 2.71 ± 0.51 s^−1^) and at 3T vs 1.5T (2.53 ± 0.48 vs 2.67 ± 0.48 s^−1^).

**Table 1:** Baseline characteristics and basic CMR measurements.

Parameter	Mean±SD or N (%)*
1.5T	447 (40.7%)
3T	651 (59.3%)
Male	668 (60.8%)
Female	430 (39.1%)
Age	51.8±17.1
Weight (kg)	80.3±17.0
Height (cm)	174.6±9.8
BSA (m²)	1.98±0.24
BMI (kg/m²)	26.3±4.9
Hct	0.43±0.05
LVEDV (mL)	169.0±67.2
LVEDVi (mL/m²)	85.0±30.1
LVSV (mL)	85.3±22.3
LVSVi (mL/m²)	43.1±9.9
LVEF (%)	53.6±13.1
HR (/min)	71.6±14.6
CO (L/min)	6.01±1.71
CI (L/min/m²)	3.04±0.77
ΔR1_blood_ (s^−1^)	2.59±0.49

Total N = 1098

*BMI* body mass index, *BSA* body surface area, *CI* cardiac index, *CMR* cardiovascular magnetic resonance, *CO* cardiac output, *Hct* hematocrit, *HR* heart rate, *LVEDV* left ventricular end-diastolic volume, *LVEDVi* left ventricular end-diastolic volume index, *LVEF* left ventricular ejection fraction, *LVSV* left ventricular stroke volume, *LVSVi* left ventricular stroke volume index, *N* number of observations

* Data are numbers (%) of cases or means ± standard deviation (SD).

### 3.2 Descriptive data analysis

**Fig. 2:**
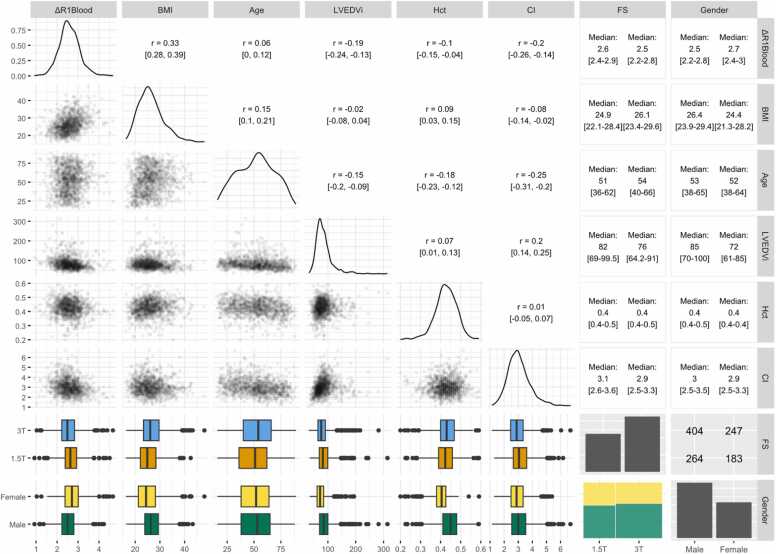
Bivariate analysis of ΔR1_blood_ and the predictor variables. Diagonal shows density plots for continuous and bar charts for factorial variables. Upper triangle shows Pearson correlation coefficients with 95% confidence intervals for continuous variables, a cross-table for the factorial variables and medians with interquartile ranges for combinations of continuous and factorial variables. Lower triangle shows scatter plots for continuous variables, a mosaic plot for the factorial variables and boxplots for combinations of factorial and continuous variables. Common x-axis for all plots in same column. Common y-axis for scatter- and boxplots in same row, density plots y-axes not shown except for ΔR1_blood_. Created using R “ggpairs” package [17]. *BMI* body mass index, *CI* cardiac index, *FS* field strength, *Hct* hematocrit, *LVEDVi* left ventricular end-diastolic volume index

Density plots for continuous variables and bar plots for factorial variables are shown in the diagonal of Fig. 2. Histograms and QQ plots for ΔR1_blood_ and all continuous predictor values are shown in the Supplementary Material and show slight to moderate deviations from normality. Bivariate analysis is shown in the upper and lower triangles of Fig. 2, with moderate correlation between ΔR1_blood_ and BMI (r = 0.33) and weak to moderate correlation between ΔR1_blood_ and all other continuous predictor variables. There was overall weak to moderate collinearity within the covariates, the largest being between CI and age (r = −0.25) and CI and LVEDVi (r = 0.2).

**Fig. 3:**
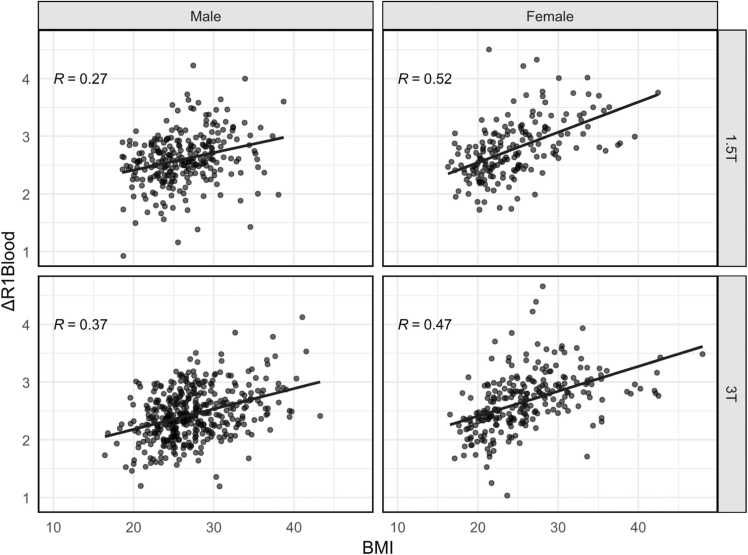
Scatter plots of ΔR1_blood_ vs body mass index (BMI) by gender and field strength.

A scatter plot of ΔR1_blood_ vs BMI, separated by gender and FS, is depicted in Fig. 3, showing stronger correlation in women than in men at both 1.5T and 3T, suggesting interaction of BMI and gender.

### 3.3 Multivariable linear regression

The initial multiple linear regression model, based on a-priori knowledge and depicted in the directed acyclical graph (Fig. 1), consisted of FS, BMI, gender, age, Hct, LVEDVi, CI, and BMI:gender with centered covariates. Stepwise pruning of variables with *P-*values >0.05 in the model was employed, eliminating Hct, and age and leading to the final model of FS, gender, BMI, LVEDVi, CI, and BMI:gender.

A histogram and QQ plot of the residuals, as well as a scatter plot of residuals vs predicted values, are shown in the Supplementary Material. While visual inspection did not suggest non-normality or heteroskedasticity, the Shapiro-Wilks and Breusch-Pagan tests were statistically highly significant (*P*-value <0.001 for both). Although this reflects mostly the large sample size, we switched to a regression with robust SE (method: HC3) for the final model shown in Table 2. The original regression without robust SE is shown in the Supplementary Material. A sensitivity analysis is given in the Supplementary Material, showing that the effects were preserved when excluding outliers based on a Cook-distance of 4/n.

**Table 2:** Multiple linear regression with robust standard errors, unstandardized coefficients.

N = 1098	Initial model		Final model		
Term	Coefficient b [95%-CI]	*P*-value	Coefficient b [95%-CI]	ηp²	*P*-value
(Intercept)	2.6374 [2.5922; 2.6827]	<0.001*	2.6345 [2.5898; 2.6791]		<0.001*
FS (3T)	−0.2222 [−0.2736; −0.1709]	<0.001*	−0.2250 [−0.2761; −0.1739]	0.05	<0.001*
BMI	0.0328 [0.0251; 0.0405]	<0.001*	0.0322 [0.0246; 0.0398]	0.13	<0.001*
Gender (female)	0.2157 [0.1589; 0.2725]	<0.001*	0.2273 [0.1745; 0.2802]	0.09	<0.001*
Age (y)	−0.0013 [−0.0029; 0.0003]	0.102			
Hct	−0.2459 [−0.7476; 0.2558]	0.337			
LVEDVi (mL/m²)	−0.0020 [−0.0029; −0.0011]	<0.001*	−0.0019 [−0.0028; −0.0010]	0.02	<0.001*
CI (L/min/m²)	−0.1065 [−0.1414; −0.0716]	<0.001*	−0.0999 [−0.1342; −0.0656]	0.04	<0.001*
BMI:gender (female)	0.0124 [0.0020; 0.0228]	0.019*	0.0123 [0.0020; 0.0227]	0.01	0.020*
Multiple R²	0.270	<0.001*	0.268		<0.001*
Adjusted R²	0.265		0.264		

**Fig. 4:**
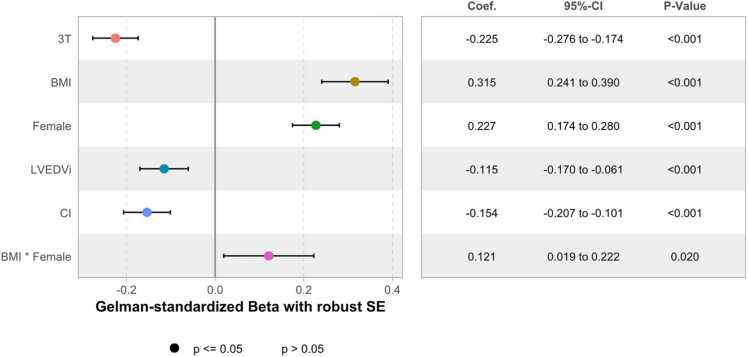
Standardized coefficient estimates (beta) for the final model with robust (HC3) standard errors (N = 1098). Continuous predictors were standardized by subtracting the mean and dividing by 2 standard deviations, following Gelman, to facilitate comparison with binary predictors [16]. Factors (FS and gender) and outcome (ΔR1_blood_) are not standardized. Robust standard errors were calculated using HC3. *BMI* body mass index, *CI* cardiac index (parameter)/confidence interval (table), *FS* field strength, *LVEDVi* left ventricular end-diastolic volume index, *SE* standard error. *P*-values not corrected for multiple testing. Created using the R “ggstats“ package

Multiple linear regression for ΔR1blood with centered covariates and robust (heteroskedasticity-consistent, method: HC3) standard errors. Final model achieved through pruning from the initial model based on the *P*-value (cutoff >0.05). Standardized coefficients in Fig. 4 and Supplementary Material

*95%-CI* 95% confidence intervals*, ηp²* partial eta squared, *BMI* body mass index, *BSA* body surface area*, CI* cardiac index*, FS* field strength*, Hct* hematocrit*, LVEDVi* left ventricular end-diastolic volume index*, SE* standard error

* Statistically significant.

**Fig. 5:**
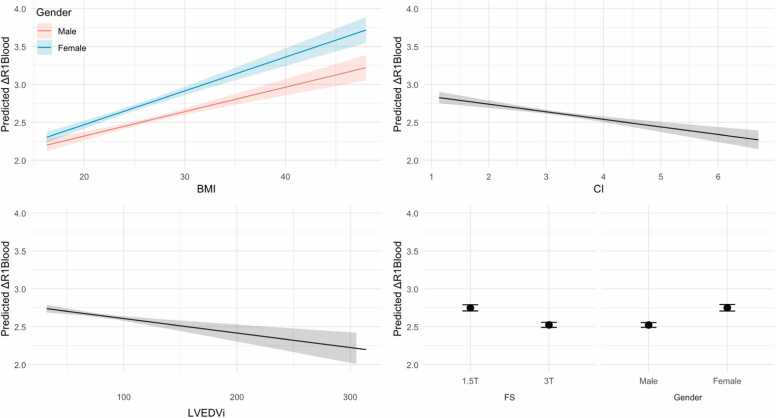
Estimated marginal means and effects based on the final model. The covariate of interest was back-transformed to uncentered values for better interpretation. All other covariates centered and held constant, effects of factors averaged. Shaded areas represent 95% confidence bands around the estimated marginal means and effects, calculated using robust standard errors (HC3). Y-axes truncated for clarity. *BMI* body mass index, *CI* cardiac index, *FS* field strength, *LVEDVi* left ventricular end-diastolic volume index

The final model with robust SE achieved an R² of 0.268 (*P*-value <0.001) (Table 2). Standardized coefficients with robust SE, using 2 standard deviations for standardization of continuous input variables according to Gelman [16], are shown in Fig. 4. BMI remained the most influential predictor with a standardized beta of 0.315, 95% confidence intervals [0.241; 0.390] and a ηp² of 0.14, corresponding to an explained variance of ΔR1_blood_ of 14%. Coefficients from fully standardized continuous input and output variables using 1 standard deviation for standardization are shown in the Supplementary Material. Estimated marginal means and effects for the final model are shown in Fig. 5.

The back-transformed multiple regression equation with uncentered covariates is:

Predicted ΔR1_blood_ = 2.6345 − 0.2250 (if 3T) + 0.2273 (if female) + (0.0322 + 0.0123 (if female))* (BMI − 26.3) − 0.0019 * (LVEDVi − 85.0) [mL/m²] − 0.0999 * (CI − 3.04) [L/min/m²].

## 4 Discussion

In this hypothesis-generating study, we found moderate dependence of BMI on ΔR1_blood_ after application of 0.15 mmol/kg in both bivariate and multivariable analyses, which was stronger in women than in men. Other variables that influenced ΔR1_blood_ in multivariable analysis were CI, LVEDVi, and FS. Neither age nor Hct was statistically significant as predictors of ΔR1_blood_ in multivariable analysis.

### 4.1 Pharmacokinetics of gadolinium

**Fig. 6:**
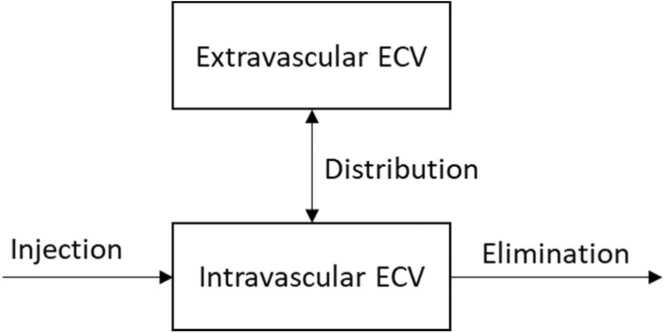
Schematic two-compartment model of gadolinium pharmacokinetics. *ECV* extracellular volume

The pharmacokinetics of gadolinium is commonly described by a two-compartment model (Fig. 6) [18]. The gadolinium plasma concentration at any given time point is therefore dependent on six main variables: the amount of gadolinium applied, the size of the central compartment, the size of the peripheral compartment, the distribution half-life, the elimination half-life, and the time of sampling.

The amount of gadolinium applied was 0.15 mmol/kg body weight, irrespective of gender and BMI. As previous research has shown an equilibrium of gadolinium between different extracellular compartments as early as 5 min after application, the distribution would be mostly finished at 15 min, followed by a steady state between compartments, and differences in distribution half-life are inconsequential to our results [19].

As gadolinium is almost entirely excreted via the kidneys, the elimination half-life varies from approximately 90 min in patients with normal renal function up to approximately 20 h in end-stage renal failure [18], [20]. This would translate to an approximately 10% lower plasma gadolinium concentration at 15 min in patients with normal renal function compared to patients with end-stage renal failure. While both obesity and female gender have been linked to lower glomerular filtration rate (GFR), the expected difference is insufficient to explain the magnitude of our observed effect [21], [22].

The time of sampling was approximately 15 min post contrast application. While the time of sampling does affect the plasma gadolinium concentration, any deviations are expected to be equally distributed among BMI and gender and therefore cannot explain our observed differences.

This leaves the sizes of the central and peripheral compartments as the most likely sources of variation in the observed ΔR1_blood_. Further differentiation would require two-compartment modeling with measurements at multiple time points after gadolinium application, which are beyond the scope of this retrospective analysis but could be implemented in a prospective trial.

### 4.2 Impact of BMI and gender on ΔR1_blood_

Among all input variables, BMI showed the strongest association with ΔR1_blood_ in both bivariate (r = 0.33, Fig. 2) and multivariable analysis (η² = 0.13, Table 2). Our theory, which is to be confirmed in prospective studies, is that this relates to a smaller volume of distribution for gadolinium per kg of body weight due to the higher percentage of total body fat in patients with higher BMI. Future studies should aim to measure the body fat percentage directly, as elevated BMI does not always imply elevated body fat but might also be due to high muscle mass.

Gender not only influenced ΔR1_blood_ directly, but significantly affected the relationship between BMI and ΔR1_blood_ in our study, with a higher regression coefficient in women in bivariate analysis (Fig. 3) and a significant interaction of BMI and gender in multiple linear regression (Table 2, Fig. 5). Previous studies have shown a curvilinear relationship between BMI and percentage of body fat, with both a higher percentage of body fat and a stronger correlation of percentage of body fat with BMI in women, but not a steeper curve [23]. The steeper curve for the regression of ΔR1_blood_ on BMI that we found in women compared to men can be explained by two facts. First, men had a higher average BMI than women in our study (26.9 ± 4.5 vs 25.3 ± 5.4 kg/m²), placing men on the flatter part of the BMI/body-fat curve. Second, the relationship between percentage of body fat and C_Gd_ is inherently non-linear, as C_Gd_ is determined by the ratio of gadolinium dose and distribution volume. Further changes in an already small distribution volume, therefore, create larger changes in C_Gd_.

### 4.3 Impact of FS on ΔR1_blood_

We found a higher ΔR1_blood_ at 1.5T compared to 3T, which is in line with previous reports of a slightly higher relaxivity of gadobutrol at 1.5T vs 3T [24]. It is worth noting in this context that the SNR and contrast-to-noise ratio (CNR) at 3T are generally higher than at 1.5T for any given ΔR1_blood_. A study using 0.15 mmol/kg gadopentetate dimeglumine showed a 65% higher SNR for infarcted myocardium and almost twice the CNR of infarcted vs remote myocardium at 3T compared to 1.5T [25]. Achieving identical ΔR1_blood_ at 1.5T and 3T is therefore probably neither necessary nor desirable for most clinical purposes, as the optimal ΔR1_blood_ might be lower at 3T than at 1.5T. Unfortunately, the optimal ΔR1_blood_ has not yet been established, and recommendations for contrast dosing in LGE imaging do not currently differ based on FS [1].

### 4.4 Impact of CI on ΔR1_blood_

We found a weak to moderate negative correlation between CI and ΔR1_blood_ in bivariate analysis (unstandardized b = −0.0999 [−0.1342; −0.0656], r = −0.20, Fig. 2) and a weak to moderate negative effect in multiple linear regression (ηp² = 0.04, *P*-value <0.001, Table 2). This is in line with anesthesiologic studies that found a negative correlation between CO and plasma sufentanyl concentrations in pigs [5]. One possible explanation is a potentially more rapid distribution and elimination of gadolinium in patients with high CI. The validity of this explanation should be tested in prospective studies with serial ΔR1_blood_ measurements at incrementing time intervals after contrast application in conjunction with GFR assessment.

### 4.5 Impact of LVEDVi on ΔR1_blood_

We found a weak to moderate negative correlation of LVEDVi with ΔR1_blood_ in bivariate analysis (r = −0.19, Fig. 2) and a weak negative effect in multiple linear regression (unstandardized b = −0.0019 [−0.0028; −0.0010], ηp² = 0.02, *P*-value <0.001, Table 2). This could be explained by increased extracellular water in patients with volume overload, which also causes left ventricular dilatation. Incorporating measures of volume overload, such as N-terminal pro-hormone brain natriuretic peptide, into future studies would enable the confirmation or rejection of this hypothesis.

### 4.6 Impact of Hct on ΔR1_blood_

As gadobutrol is strictly extracellular, the blood-pool C_Gd_ and therefore ΔR1_blood_ should be negatively correlated to Hct. Contrary to theoretical assumptions, we found only weak negative correlation of Hct and ΔR1_blood_ on bivariate analysis (r = −0,1) and no statistically or clinically significant association with ΔR1_blood_ in the multiple linear regression. The reason for this evades our current understanding and warrants further study, as current formulas for the calculation of myocardial ECV incorporate both Hct and ΔR1_blood_ due to the presumed interdependence [19].

### 4.7 Impact of age on ΔR1_blood_

Age showed only weak correlation to ΔR1_blood_ in bivariate analysis (r = 0.06) and no statistically or clinically significant effect in multiple linear regression. This is in line with previous findings of reduced total body water, but mostly unchanged extracellular body water in older patients [4].

### 4.8 Implications for clinical practice

Our study shows considerable variation in ΔR1_blood_, a surrogate of blood-pool C_Gd_ and a measure of the T1 shortening effect of gadolinium, at the time of late enhancement imaging. Unintended variations in effective gadolinium concentration are undesirable, as underdosing directly affects image quality and sensitivity [26], whereas higher than necessary gadolinium concentrations should be avoided for economic, environmental and medical reasons. Gadolinium is costly, an environmental hazard, and can deposit in the body [27], [9], [28]. Although the risk of nephrogenic systemic fibrosis is exceedingly rare with the use of the current generation of macrocyclic contrast agents, the lowest dose possible to answer the diagnostic question should be used, especially in light of detectable gadolinium deposition in the brain of yet unknown significance [29].

We were able to explain ∼27% of the total variation in ΔR1_blood_ by a linear model consisting of FS, BMI, gender, CI, LVEDVi, and BMI:gender. At 1.5T, a male with a BMI of 18, LVESVi of 130, and CI of 4 would have a predicted ΔR1_blood_ of 2.19, while a female with a BMI of 40, LVESVi of 60, and CI of 2 would have a predicted ΔR1_blood_ of 3.62, translating to a 65% difference in blood-pool C_Gd_. While our data should not be used to change existing dosing protocols, we show potential for more individualized dosing schemes to be evaluated in future prospective trials.

## 5 Limitations

There are several limitations of this analysis, most of which are related to its retrospective nature, due to which our results should be considered hypothesis-generating.

First, we did not measure C_Gd_ directly but ΔR1_blood_ as a surrogate. Nevertheless, ΔR1_blood_ represents the contrast-enhancing effect of gadolinium and can therefore be considered as relevant for imaging purposes as C_gd_ itself. Second, while ΔR1_blood_ represents the contrast-enhancing effect of gadolinium on the blood-pool, image contrast depends on the combined effect on blood-pool, myocardium, and scar. Prospective studies on the effects of BMI and body composition on the contrast between blood-pool, healthy myocardium, and scar/fibrosis on LGE imaging are therefore necessary. Third, renal function was not assessed systematically and therefore not analyzed. As gadobutrol is excreted solely via the kidneys, renal function has a direct effect on its plasma half-life and therefore its plasma concentration at any given time point [20]. Fourth, the time from contrast application to post contrast T1 mapping was not assessed. Again, this directly influences the plasma C_Gd_. Fifth, some patients received the total dose of gadobutrol at once, while others received it in two or more boluses (stress and rest perfusion).

With the possible exception of kidney function, these aforementioned confounders would be expected to be independent of the predictor variables and therefore equally distributed among their range. The effect of kidney function on ΔR1_blood_ and image contrast should be examined in future studies. With regards to FS, confounding seems possible because of differing contrast dosing schemes, as stated in Section 2. Therefore, the difference of ΔR1_blood_ between 1.5T and 3T should be interpreted with caution.

Furthermore, we examined ΔR1_blood_ at the time of late enhancement imaging, where the volume of distribution can be equated to the total body ECV. Our results can therefore not be interpolated to dosing for first-pass perfusion or contrast angiography, where the volume of distribution is rather related to the intravascular ECV.

Lastly, BMI is an imperfect surrogate of the percentage of body fat. Measurements of body fat are usually not available at the time of CMR imaging, and it is unclear whether its measurement would add sufficient clinical value to offset its cost. Future studies should therefore assess the relationship between BMI, body fat percentage, contrast agent distribution, and image contrast.

## 6 Conclusion

ΔR1_blood_, a measurement of gadolinium contrast enhancement in the blood-pool and a surrogate of plasma C_Gd_ at the time of late enhancement imaging, showed a moderate association with BMI, FS, and gender and weak association with LVEDVi and CI. Our hypothesis-generating findings invite further studies to assess the need for individualized gadolinium dosing incorporating body composition, FS, and cardiac function.

## Funding

S.K. and P.D. were supported by a grant from Philips Healthcare. S.K. received funding from the 10.13039/100010447DZHK (German Center for Cardiovascular Research) and the 10.13039/501100002347BMBF (German Ministry of Education and Research). S.K. and R.B. were funded by the 10.13039/501100001659Deutsche Forschungsgemeinschaft (DFG, German Research Foundation)—SFB-1470-B06. J.L. was funded by the Kaltenbach dissertational grant of the 10.13039/501100005971Deutsche Herzstiftung.

## Author contributions

**Henryk Dreger:** Supervision, Writing – review & editing, Resources. **Jeffrey Ji-Peng Li:** Writing – review & editing. **Patrick Doeblin:** Writing – original draft, Visualization, Conceptualization, Methodology, Writing – review & editing, Formal analysis. **Sebastian Kelle:** Software, Writing – review & editing, Resources, Supervision. **Wensu Chen:** Investigation, Data curation. **Shing Ching:** Formal analysis, Data curation. **Stefanie Maria Werhahn:** Writing – review & editing. **Natalia Solowjowa:** Writing – review & editing. **Rebecca Elisabeth Beyer:** Writing – review & editing. **Christian Stehning:** Writing – review & editing, Methodology, Validation. **Misael Estepa:** Writing – review & editing.

## Ethics approval and consent

The study was approved by the local ethics committee (EA2/073/21) with a waiver of informed consent.

## Consent for publication

Not applicable.

## Declaration of competing interests

S.K. received funding from the DZHK (German Centre for Cardiovascular Research), by the BMBF (German Ministry of Education and Research), personal fees from Servier, a grant from Philips Healthcare, and lecture honoraria from Medis, NL. P.D. received travel subsidies from Philips Healthcare. C.S. is an employee of Philips Healthcare.

## References

[1] Kramer C.M., Barkhausen J., Bucciarelli-Ducci C., Flamm S.D., Kim R.J., Nagel E. (2020). Standardized cardiovascular magnetic resonance imaging (CMR) protocols: 2020 update. J Cardiovasc Magn Reson.

[2] Ritz P., Vol S., Berrut G., Tack I., Arnaud M.J., Tichet J. (2008). Influence of gender and body composition on hydration and body water spaces. Clin Nutr.

[3] Ohashi Y., Joki N., Yamazaki K., Kawamura T., Tai R., Oguchi H. (2018). Changes in the fluid volume balance between intra- and extracellular water in a sample of Japanese adults aged 15-88 yr old: a cross-sectional study. Am J Physiol Ren Physiol.

[4] Schoeller D.A. (1989). Changes in total body water with age. Am J Clin Nutr.

[5] Birkholz T., Leuthold C., Schmidt J., Ihmsen H., Schuttler J., Jeleazcov C. (2018). Influence of cardiac output on the pharmacokinetics of sufentanil in anesthetized pigs. Anesthesiology.

[6] Montalt-Tordera J., Quail M., Steeden J.A., Muthurangu V. (2021). Reducing contrast agent dose in cardiovascular MR angiography with deep learning. J Magn Reson Imaging.

[7] Monti C.B., Codari M., Cozzi A., Ali M., Saggiante L., Sardanelli F. (2020). Image quality of late gadolinium enhancement in cardiac magnetic resonance with different doses of contrast material in patients with chronic myocardial infarction. Eur Radiol Exp.

[8] Doeblin P., Steinbeis F., Witzenrath M., Hashemi D., Chen W., Weiss K.J. (2023). Half-dose versus single-dose gadobutrol for extracellular volume measurements in cardiac magnetic resonance. J Cardiovasc Dev Dis.

[9] Reiter T., Ritter O., Prince M.R., Nordbeck P., Wanner C., Nagel E. (2012). Minimizing risk of nephrogenic systemic fibrosis in cardiovascular magnetic resonance. J Cardiovasc Magn Reson.

[10] Chen W., Doeblin P., Al-Tabatabaee S., Klingel K., Tanacli R., Weiß K.J. (2022). Synthetic extracellular volume in cardiac magnetic resonance without blood sampling: a reliable tool to replace conventional extracellular volume. Circ Cardiovasc Imaging.

[11] Messroghli D.R., Radjenovic A., Kozerke S., Higgins D.M., Sivananthan M.U., Ridgway J.P. (2004). Modified Look-Locker inversion recovery (MOLLI) for high-resolution T1 mapping of the heart. Magn Reson Med.

[12] Textor J., van der Zander B., Gilthorpe M.S., Liskiewicz M., Ellison G.T. (2016). Robust causal inference using directed acyclic graphs: the R package 'dagitty'. Int J Epidemiol.

[13] Cohen J. (1992). A power primer. Psychol Bull.

[14] Cohen J. (1988). Statistical power analysis for the behavioral sciences.

[15] MacKinnon J.G., White H. (1985). Some heteroskedasticity-consistent covariance matrix estimators with improved finite sample properties. J Econ.

[16] Gelman A. (2008). Scaling regression inputs by dividing by two standard deviations. Stat Med.

[17] Emerson J.W., A. GW, Barret S., Jason C., Dianne C., Heike H. (2013). The generalized Pairs plot. J Comput Graph Stat.

[18] Staks T., Schuhmann-Giampieri G., Frenzel T., Weinmann H.J., Lange L., Platzek J. (1994). Pharmacokinetics, dose proportionality, and tolerability of gadobutrol after single intravenous injection in healthy volunteers. Invest Radiol.

[19] Ugander M., Oki A.J., Hsu L.Y., Kellman P., Greiser A., Aletras A.H. (2012). Extracellular volume imaging by magnetic resonance imaging provides insights into overt and sub-clinical myocardial pathology. Eur Heart J.

[20] Tombach B., Bremer C., Reimer P., Schaefer R.M., Ebert W., Geens V. (2000). Pharmacokinetics of 1M gadobutrol in patients with chronic renal failure. Invest Radiol.

[21] Lu J.L., Molnar M.Z., Naseer A., Mikkelsen M.K., Kalantar-Zadeh K., Kovesdy C.P. (2015). Association of age and BMI with kidney function and mortality: a cohort study. Lancet Diabetes Endocrinol.

[22] Fenton A., Montgomery E., Nightingale P., Peters A.M., Sheerin N., Wroe A.C. (2018). Glomerular filtration rate: new age- and gender- specific reference ranges and thresholds for living kidney donation. BMC Nephrol.

[23] Meeuwsen S., Horgan G.W., Elia M. (2010). The relationship between BMI and percent body fat, measured by bioelectrical impedance, in a large adult sample is curvilinear and influenced by age and sex. Clin Nutr.

[24] Rohrer M., Bauer H., Mintorovitch J., Requardt M., Weinmann H.J. (2005). Comparison of magnetic properties of MRI contrast media solutions at different magnetic field strengths. Invest Radiol.

[25] Klumpp B., Fenchel M., Hoevelborn T., Helber U., Scheule A., Claussen C. (2006). Assessment of myocardial viability using delayed enhancement magnetic resonance imaging at 3.0 Tesla. Invest Radiol.

[26] Kim R.J., Albert T.S., Wible J.H., Elliott M.D., Allen J.C., Lee J.C. (2008). Performance of delayed-enhancement magnetic resonance imaging with gadoversetamide contrast for the detection and assessment of myocardial infarction: an international, multicenter, double-blinded, randomized trial. Circulation.

[27] Dekker H.M., Stroomberg G.J., Van der Molen A.J., Prokop M. (2024). Review of strategies to reduce the contamination of the water environment by gadolinium-based contrast agents. Insights Imaging.

[28] Gulani V., Calamante F., Shellock F.G., Kanal E., Reeder S.B., International Society for Magnetic Resonance in Medicine (2017). Gadolinium deposition in the brain: summary of evidence and recommendations. Lancet Neurol.

[29] American College of Radiology. ACR manual on contrast media [Internet]. 2024 [cited 2025 Jul 1]. Available from: https://www.acr.org/Clinical-Resources/Clinical-Tools-and-Reference/Contrast-Manual.

