# Supplementary material for: ΔR1blood, a surrogate of blood-pool gadolinium concentration, is related to body mass index, gender, left ventricular end-diastolic volume index, cardiac index, and field strength at cardiac magnetic resonance late enhancement imaging
Source: J Cardiovasc Magn Reson. 2025 Jun 25;27(2):101929. doi: 10.1016/j.jocmr.2025.101929 (PMC12745147; doi:10.1016/j.jocmr.2025.101929)
Supplement: Supplementary file 1 — Supplementary material [file mmc1.docx]

Contents

[Histograms and QQ-Plots of continuous variables 2](#_Toc199608180)

[Multiple linear regression with non-robust standard errors 5](#_Toc199608181)

[Parameter estimates 5](#_Toc199608182)

[Residual analysis 6](#_Toc199608183)

[Fully standardized multiple linear regression with robust standard errors 8](#_Toc199608184)

[Multiple linear regression with robust standard errors and outliers excluded 8](#_Toc199608185)

[Lasso-Regression 11](#_Toc199608186)

# Histograms and QQ-Plots of continuous variables


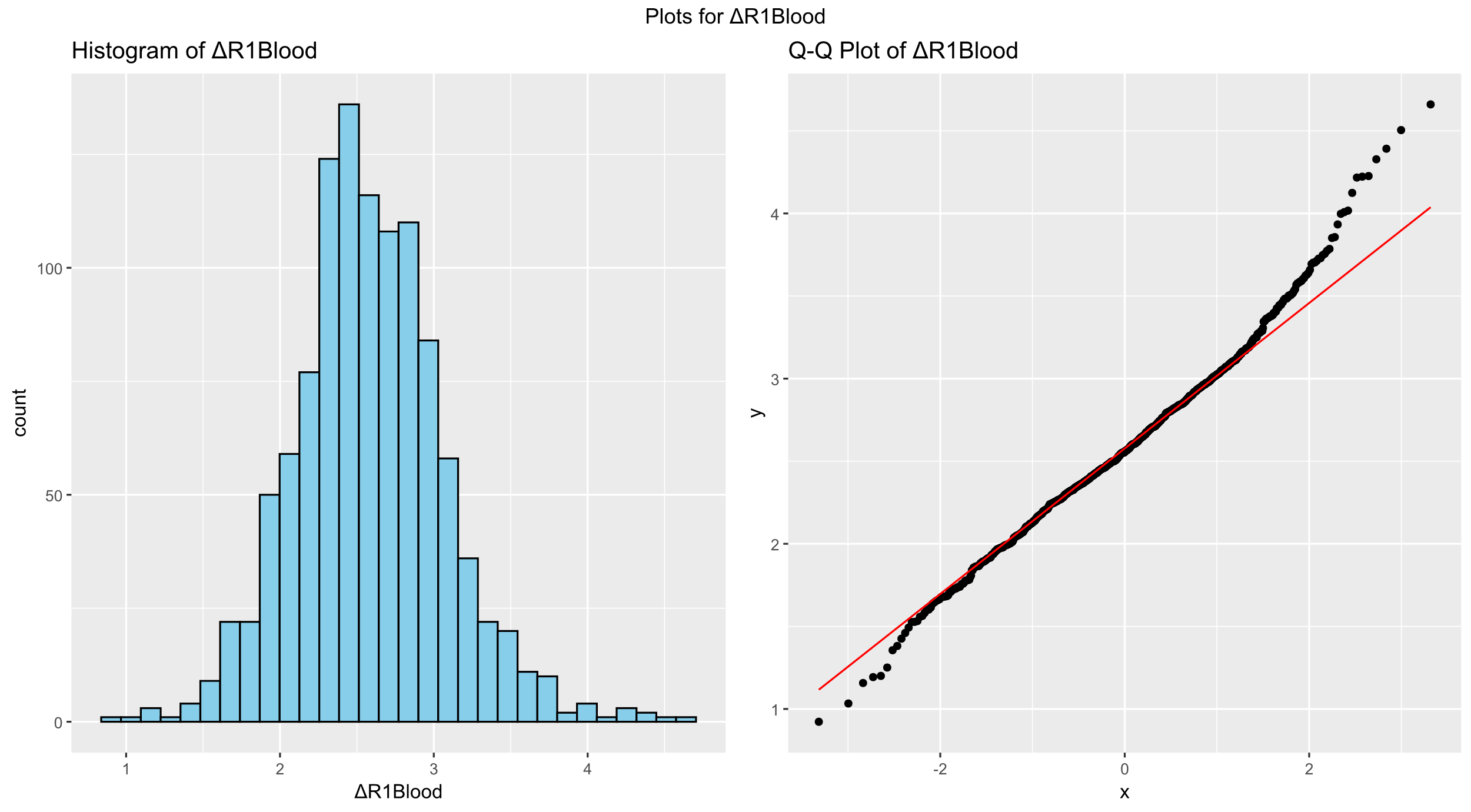


Supplementary Figure 1. Histogram and QQ-Plot for ΔR1_blood_ in s^-1^.


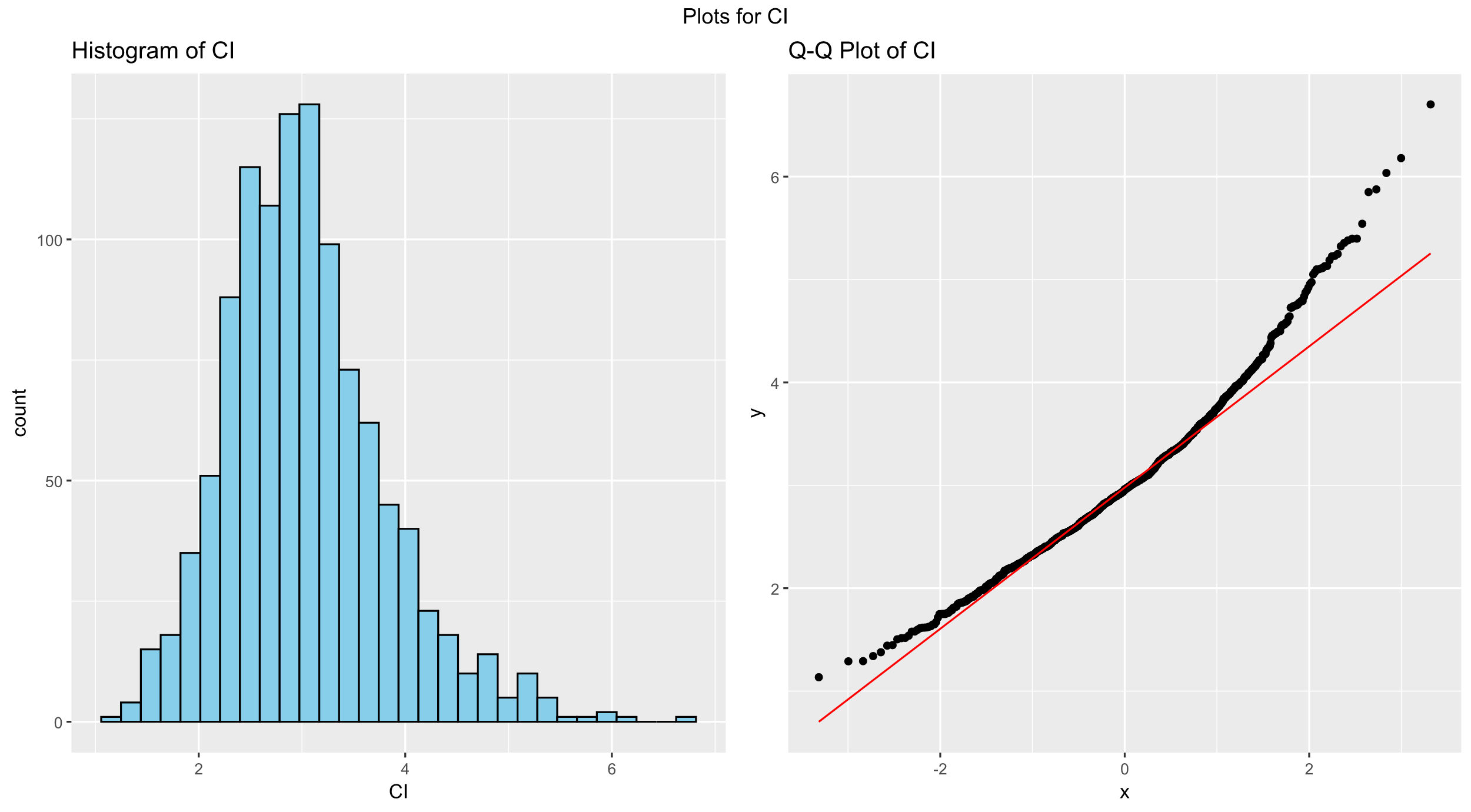


Supplementary Figure 2. Histogram and QQ-Plot for Cardiac Index (CI) in l/min/m².


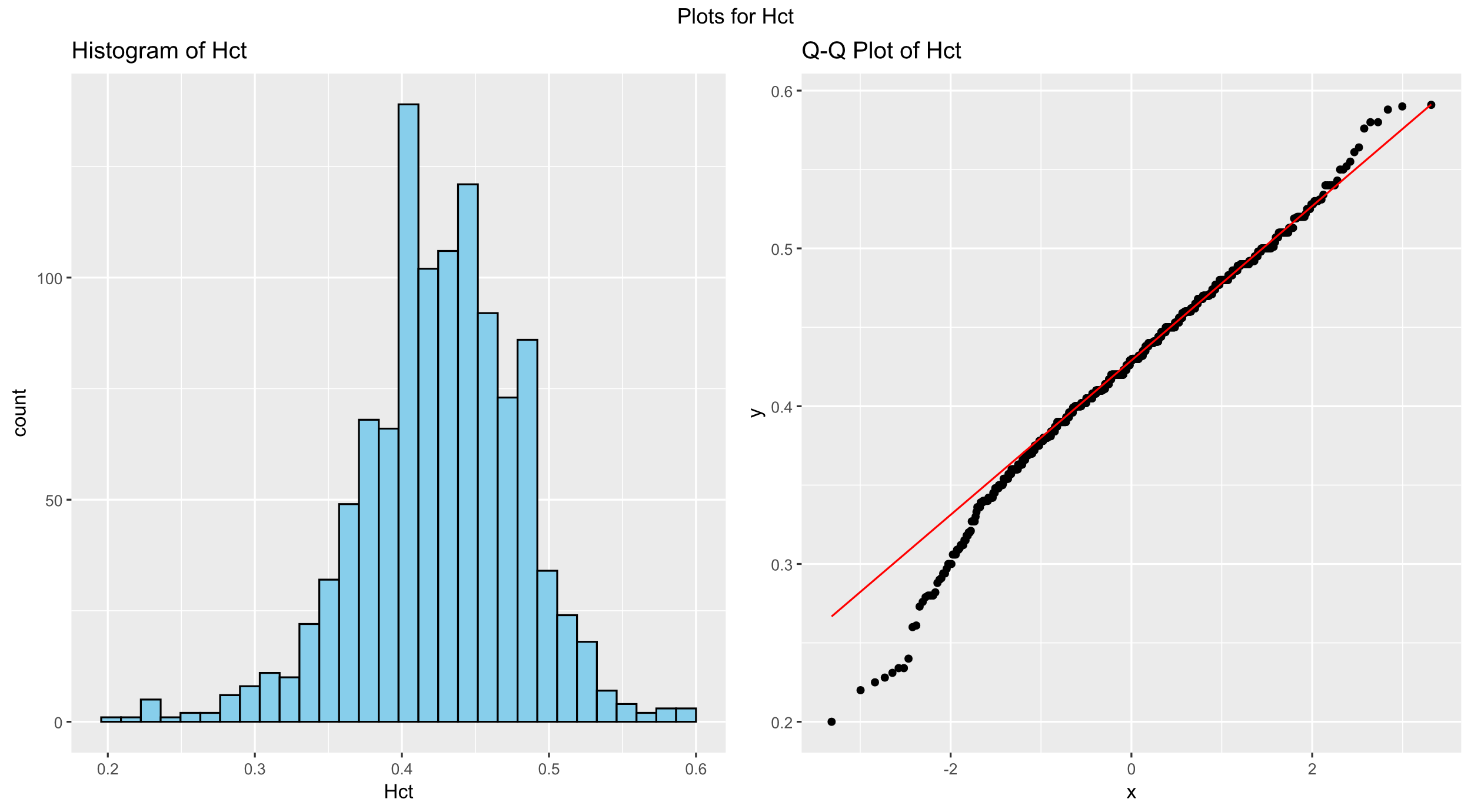


Supplementary Figure 3. Histogram and QQ-Plot for Hematocrit (Hct).


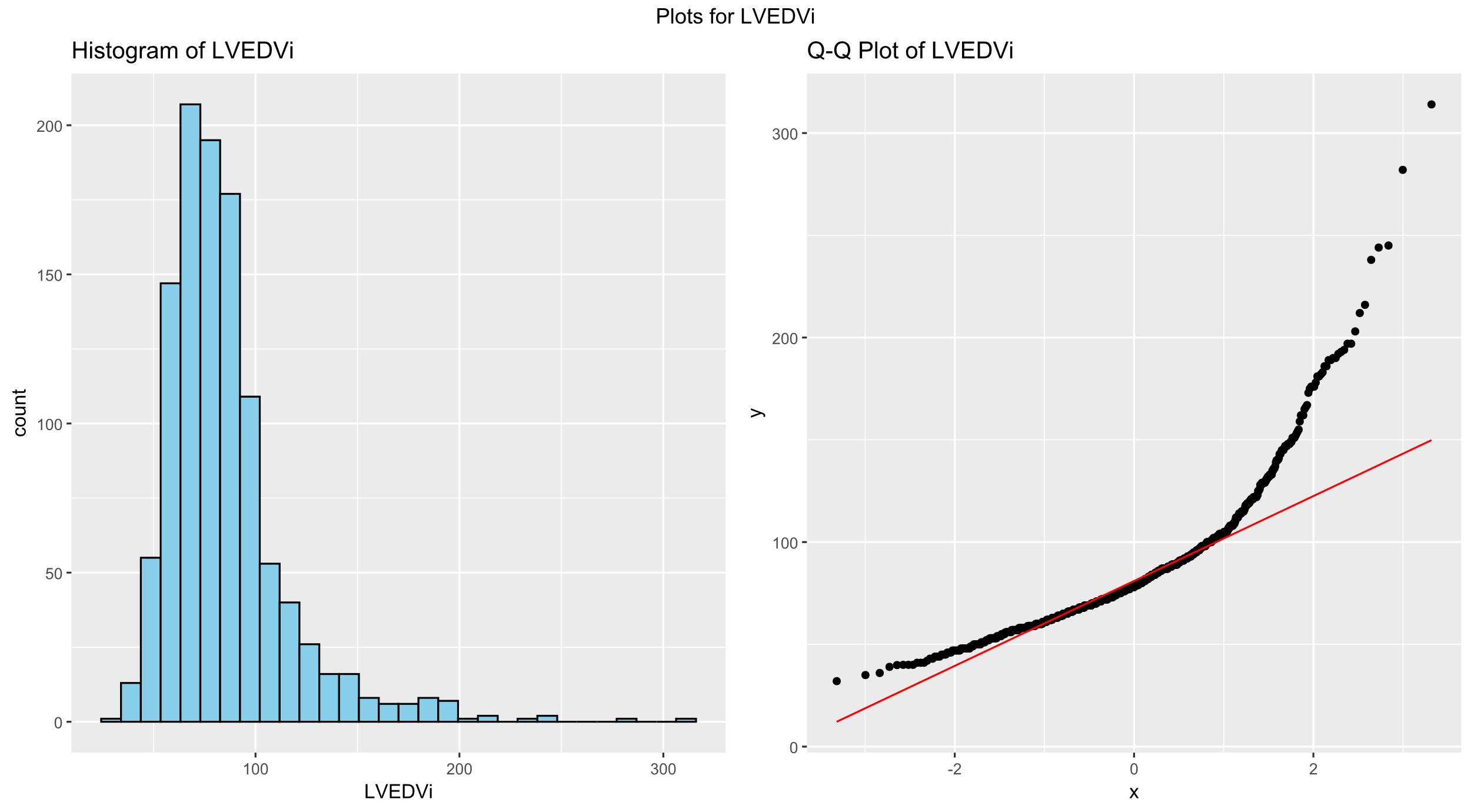


Supplementary Figure 4. Histogram and QQ-Plot for Left Ventricular End-Diastolic Volume index (LVEDVi) in ml/m².


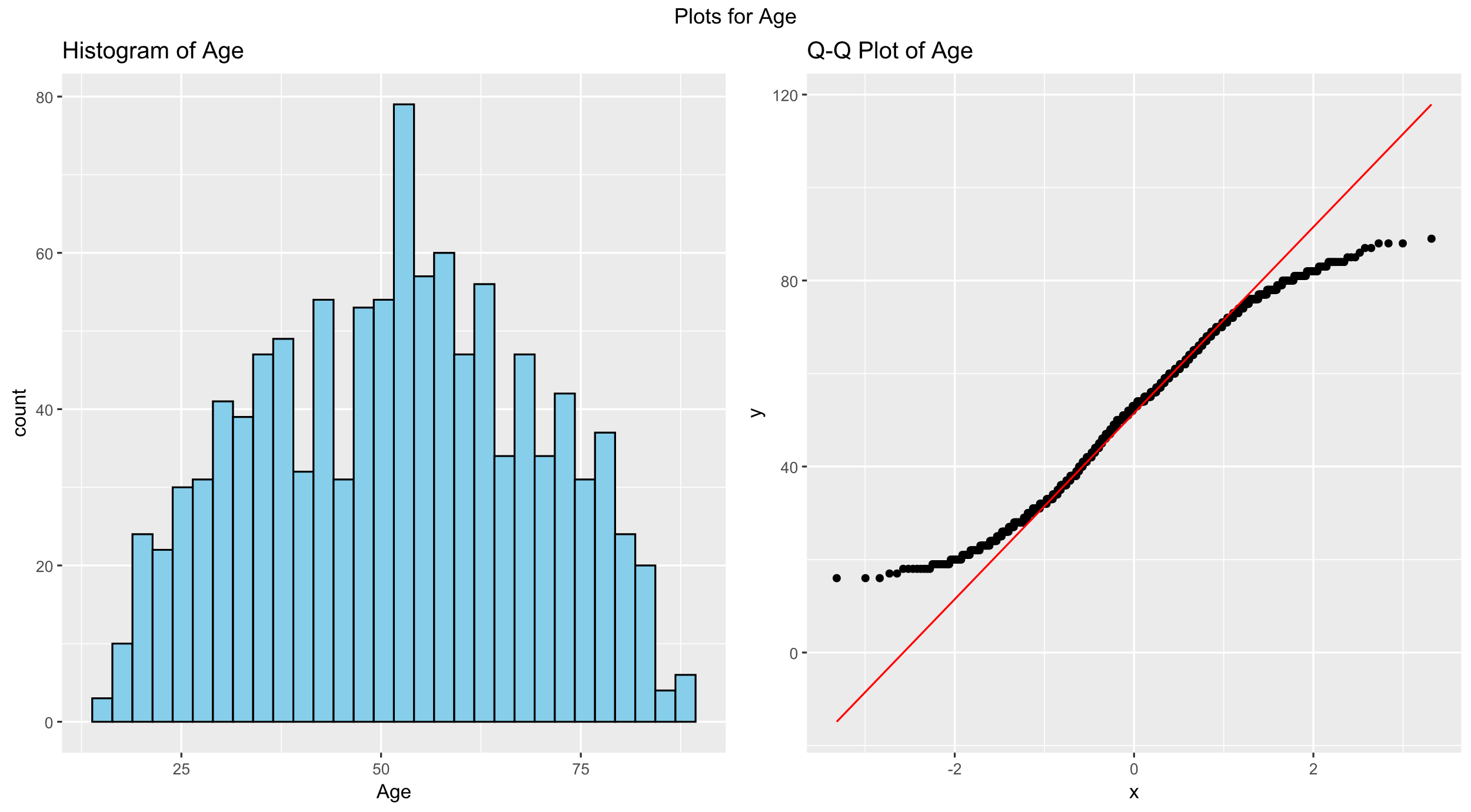


Supplementary Figure 5. Histogram and QQ-Plot for Age (years).


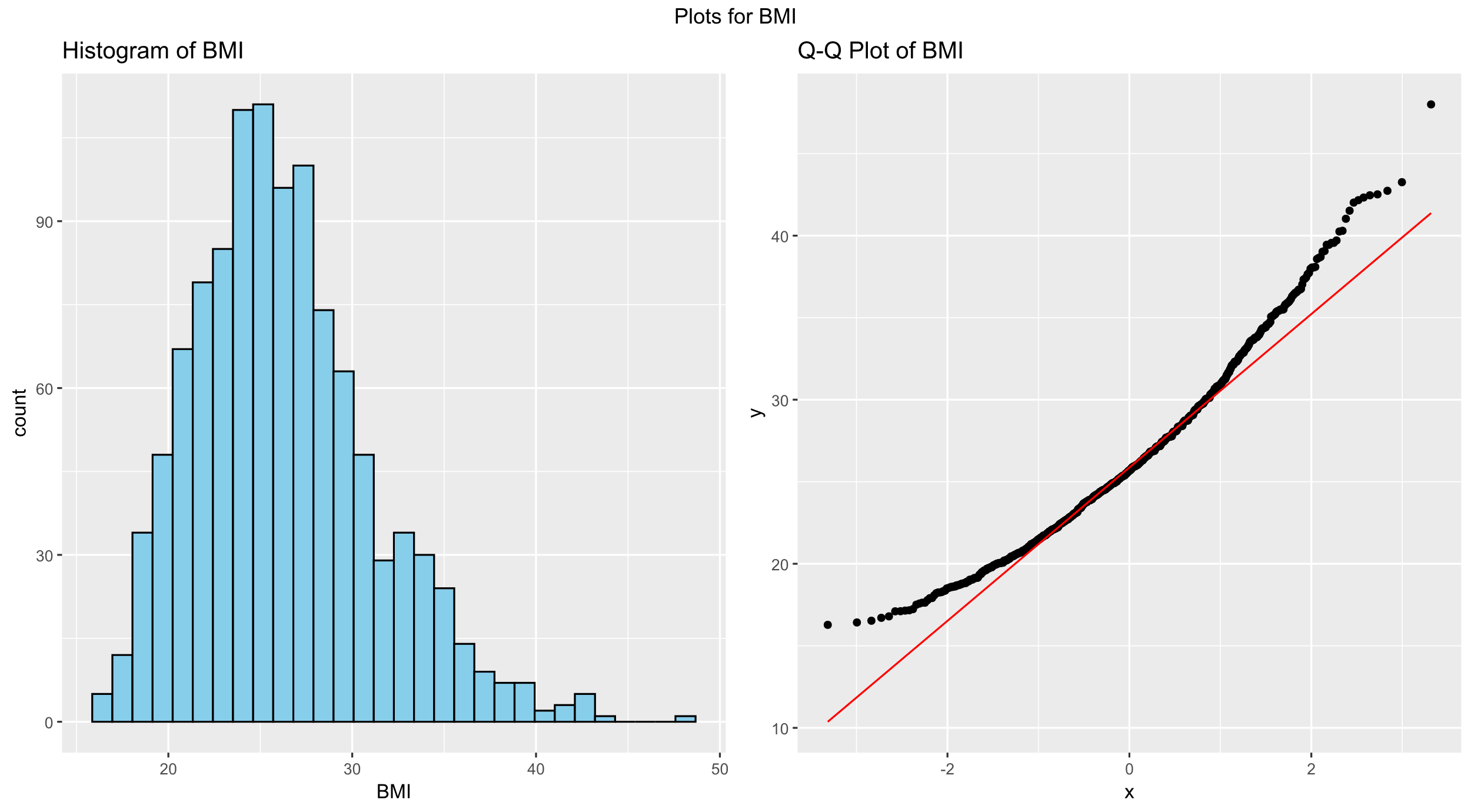


Supplementary Figure 6. Histogram and QQ-Plot for Body Mass Index (BMI).

Multiple linear regression with non-robust standard errors

## Parameter estimates

Supplementary Table 1. Parameter estimates for multiple linear regression with non-robust standard errors

| **N=1098** | **Initial Model** | |  | **Final Model** |  |  |
| --- | --- | --- | --- | --- | --- | --- |
| **Term** | **Estimate [95%-CI]** | ***P*-Value** |  | **Estimate [95%-CI]** | ***P*-Value** |  |
| (Intercept) | 2.6374 [2.5921; 2.6828] | <0.001* |  | 2.6345 [2.5895; 2.6794] | <0.001* |  |
| FS[3T] | -0.2222 [-0.2736; -0.1708] | <0.001* |  | -0.2250 [-0.2762; -0.1737] | <0.001* |  |
| BMI | 0.0328 [0.0256; 0.0400] | <0.001* |  | 0.0322 [0.0251; 0.0393] | <0.001* |  |
| Gender[Female] | 0.2157 [0.1586; 0.2728] | <0.001* |  | 0.2273 [0.1745; 0.2801] | <0.001* |  |
| Age[years] | -0.0013 [-0.0029; 0.0002] | 0.092 |  |  |  |  |
| Hct | -0.2459 [-0.7502; 0.2584] | 0.338 |  |  |  |  |
| LVEDVi [ml/m²] | -0.0020 [-0.0029; -0.0011] | <0.001* |  | -0.0019 [-0.0028; -0.0011] | <0.001* |  |
| CI [l/min/m²] | -0.1065 [-0.1403; -0.0727] | <0.001* |  | -0.0999 [-0.1329; -0.0669] | <0.001* |  |
| BMI:Gender[Female] | 0.0124 [0.0022; 0.0226] | 0.017 |  | 0.0123 [0.0021; 0.0226] | 0.0179* |  |
| Multiple R² | 0.270 | <0.001* |  | 0.268 | <0.001* |  |
| Adjusted R² | 0.265 |  |  | 0.264 |  |  |

Multiple linear regression for ΔR1_blood._ with centered covariates. Final model achieved through pruning from the initial model based on the *P*-value (cutoff >0.05). BMI body mass index, BSA body surface area, CI cardiac index, CO confidence interval, FS field strength, Hct hematocrit, LVEDVi left ventricular end-diastolic volume index. *statistically significant

## Residual analysis


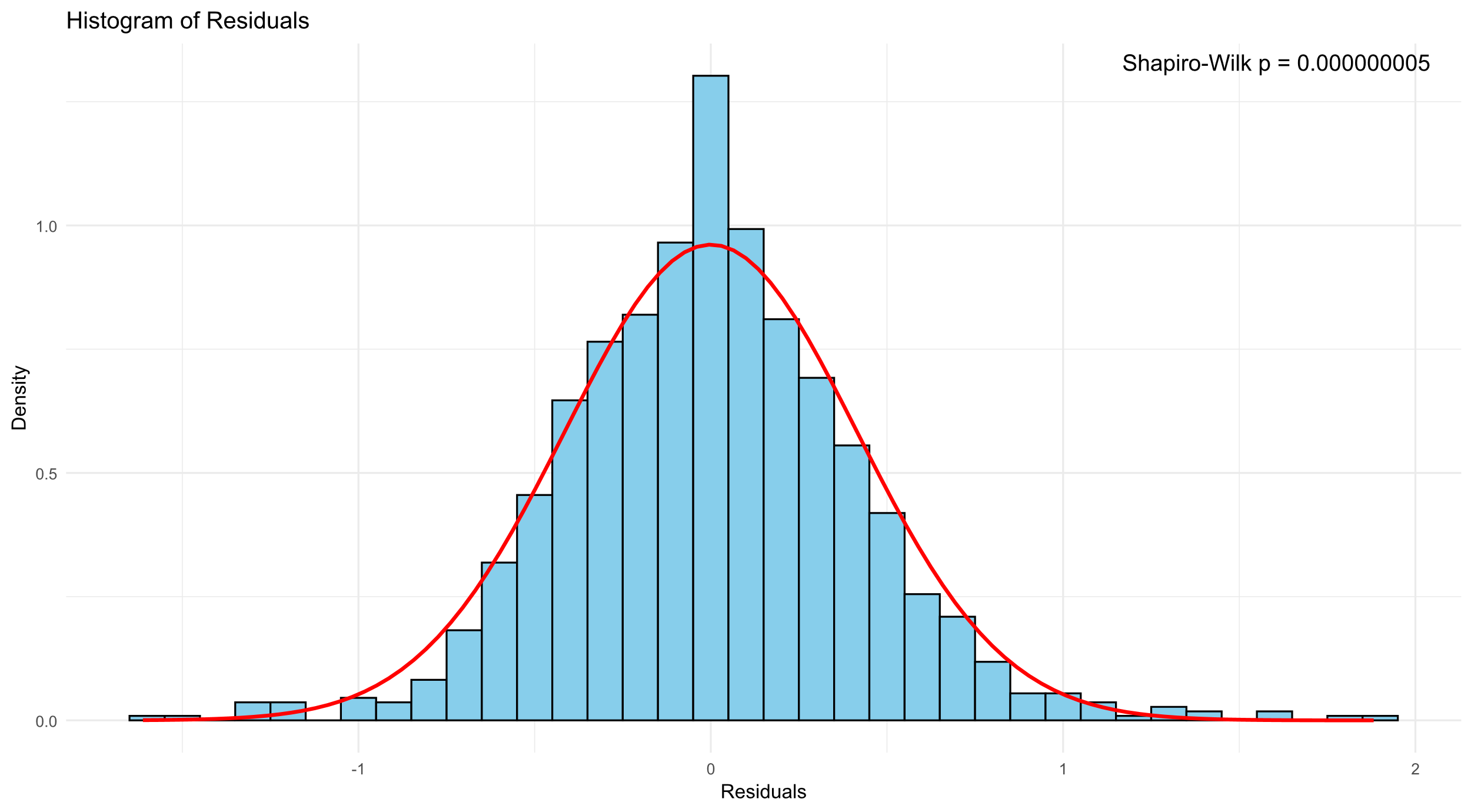


Supplementary Figure 7. Histogram of residuals of the final model, showing slight deviation from the normal curve.


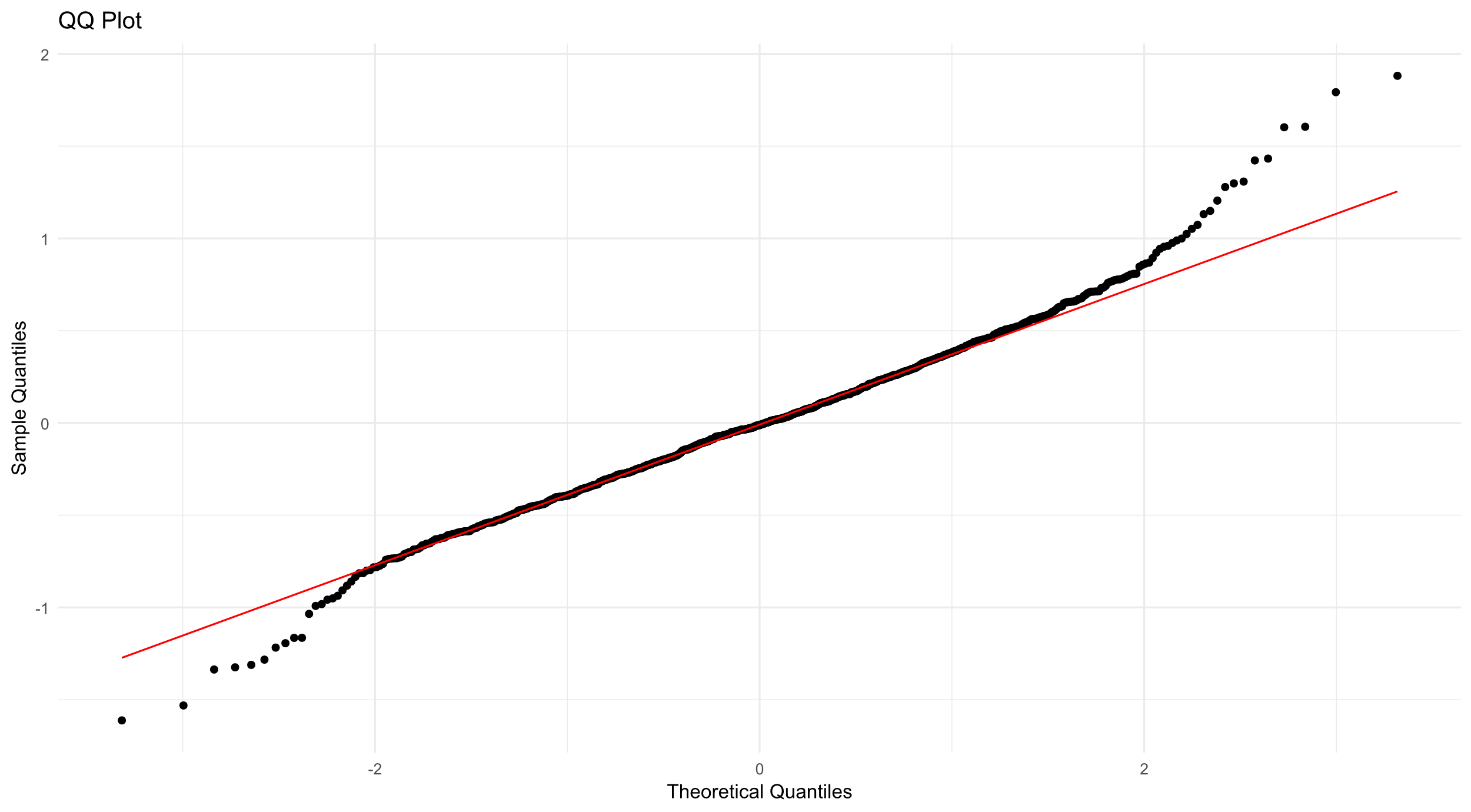


Supplementary Figure 8. Q-Q-Plot of the residuals of the final model showing slight deviations from the line of identity due to outliers.


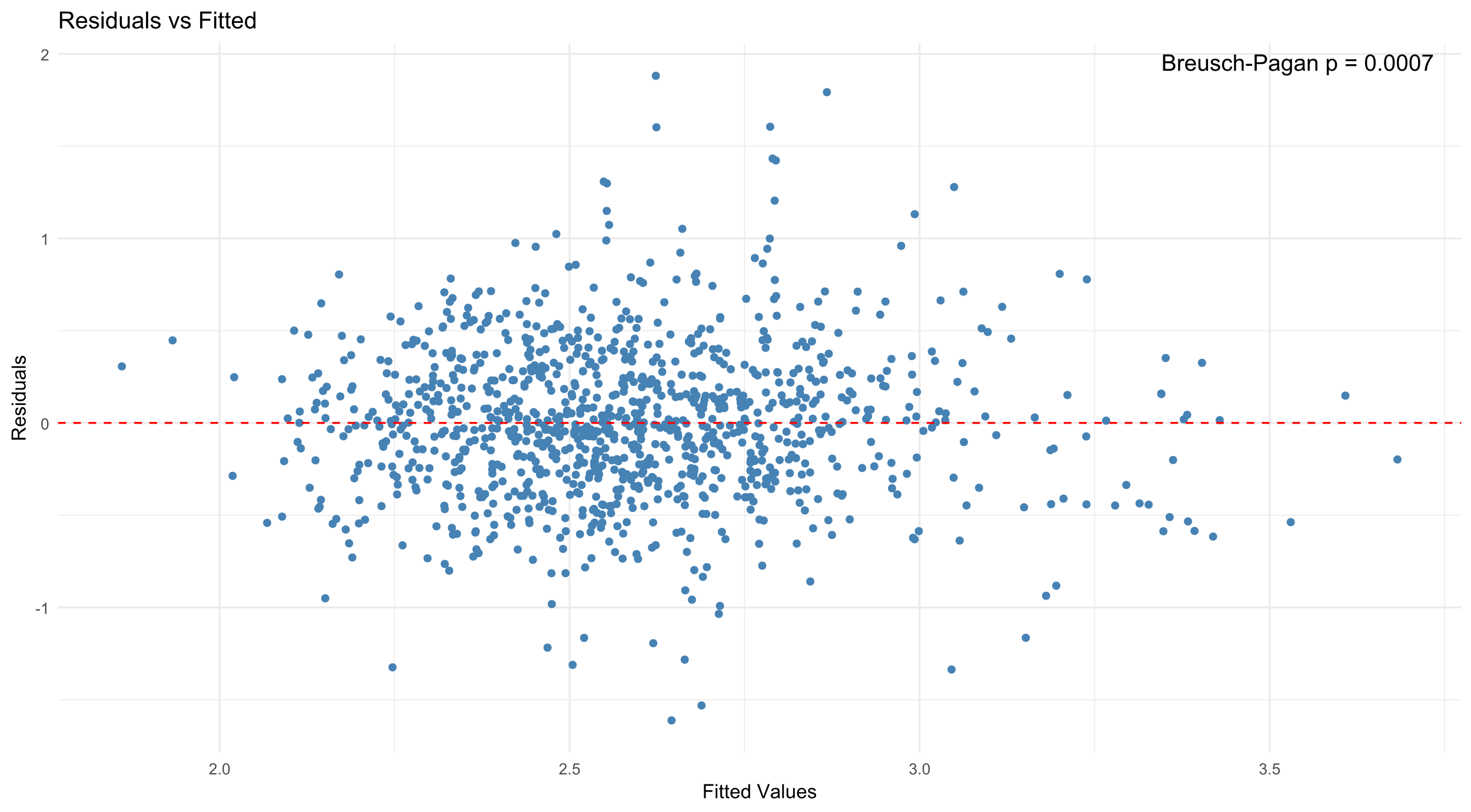


Supplementary Figure 9. Residuals versus fitted values plot for the final model, showing slight heteroscedasticity.

# Fully standardized multiple linear regression with robust standard errors

Supplementary Table 2. Fully standardized multiple linear regression with robust standard errors.

| **Term** | **Initial Model** | **Final Model** |
| --- | --- | --- |
| (Intercept) | 0.107 [0.014; 0.201] | 0.101 [0.009; 0.193] |
| FS[3T] | -0.458 [-0.564; -0.352] | -0.464 [-0.569; -0.358] |
| BMI | 0.331 [0.253; 0.409] | 0.325 [0.248; 0.402] |
| Gender[Female] | 0.445 [0.328; 0.562] | 0.469 [0.360; 0.578] |
| Age[years] | -0.047 [-0.103; 0.009] |  |
| Hct | -0.028 [-0.084; 0.029] |  |
| LVEDVi [ml/m²] | -0.124 [-0.181; -0.067] | -0.119 [-0.175; -0.063] |
| CI [l/min/m²] | -0.169 [-0.224; -0.114] | -0.159 [-0.213; -0.104] |
| BMI:Gender[F] | 0.126 [0.021; 0.230] | 0.125 [0.020; 0.229] |

N=1098. Fully standardized multiple linear regression for ΔR1blood with centered covariates and robust (heteroskedasticity-consistent, method: HC3) standard errors. Continuous predictors and ΔR1_blood_ were standardized by subtracting the mean and dividing by 1 standard deviation. BMI body mass index, BSA body surface area, CI cardiac index, CO confidence interval, F female, FS field strength, Hct hematocrit, LVEDVi left ventricular end-diastolic volume index,

# Multiple linear regression with robust standard errors and outliers excluded

Supplementary Table 3. Multiple linear regression for ΔR1_blood_ with robust standard errors and outliers excluded.

| **N=1030** | **Initial Model with robust SE** | |  | **Final Model with robust SE** |  |  |
| --- | --- | --- | --- | --- | --- | --- |
| **Term** | **Estimate [95%-CI]** | ***P*-Value** |  | **Estimate [95%-CI]** | ***P*-Value** |  |
| (Intercept) | 2.6455 [2.6084; 2.6827] | <0.001* |  | 2.6410 [2.6039; 2.6780] | <0.001* |  |
| FS[3T] | -0.2256 [-0.2686; -0.1826] | <0.001* |  | -0.2292 [-0.2720; -0.1864] | <0.001* |  |
| BMI | 0.0301 [0.0241; 0.0361] | <0.001* |  | 0.0294 [0.0235; 0.0353] | <0.001* |  |
| Gender[Female] | 0.2053 [0.1585; 0.2522] | <0.001* |  | 0.2212 [0.1776; 0.2648] | <0.001* |  |
| Age[years] | -0.0012 [-0.0025; 0.0001] | 0.067 |  |  |  |  |
| Hct | -0.3506 [-0.7752; 0.0739] | 0.106 |  |  |  |  |
| LVEDVi [ml/m²] | -0.0023 [-0.0030; -0.0015] | <0.001* |  | -0.0022 [-0.0029; -0.0014] | <0.001* |  |
| CI [l/min/m²] | -0.0983 [-0.1258; -0.0709] | <0.001* |  | -0.0925 [-0.1196; -0.0654] | <0.001* |  |
| BMI:Gender[Female] | 0.0193 [0.0110; 0.0277] | <0.001* |  | 0.0192 [0.0108; 0.0275] | <0.001* |  |
| Multiple R² | 0.343 | <0.001* |  | 0.340 | <0.001* |  |
| Adjusted R² | 0.338 |  |  | 0.336 |  |  |

Multiple linear regression for ΔR1_blood_ with outliers excluded, based on Cooks-distance with a threshold of 4/N = 0.00388, centered covariates and robust standard errors (method: HC3). BMI body mass index, BSA body surface area, CI cardiac index, CO confidence interval, FS field strength, HC heteroskedasticity consistent, Hct hematocrit, LVEDVi left ventricular end-diastolic volume index, SE standard error. *statistically significant

# Lasso-Regression

Using R library glmnet


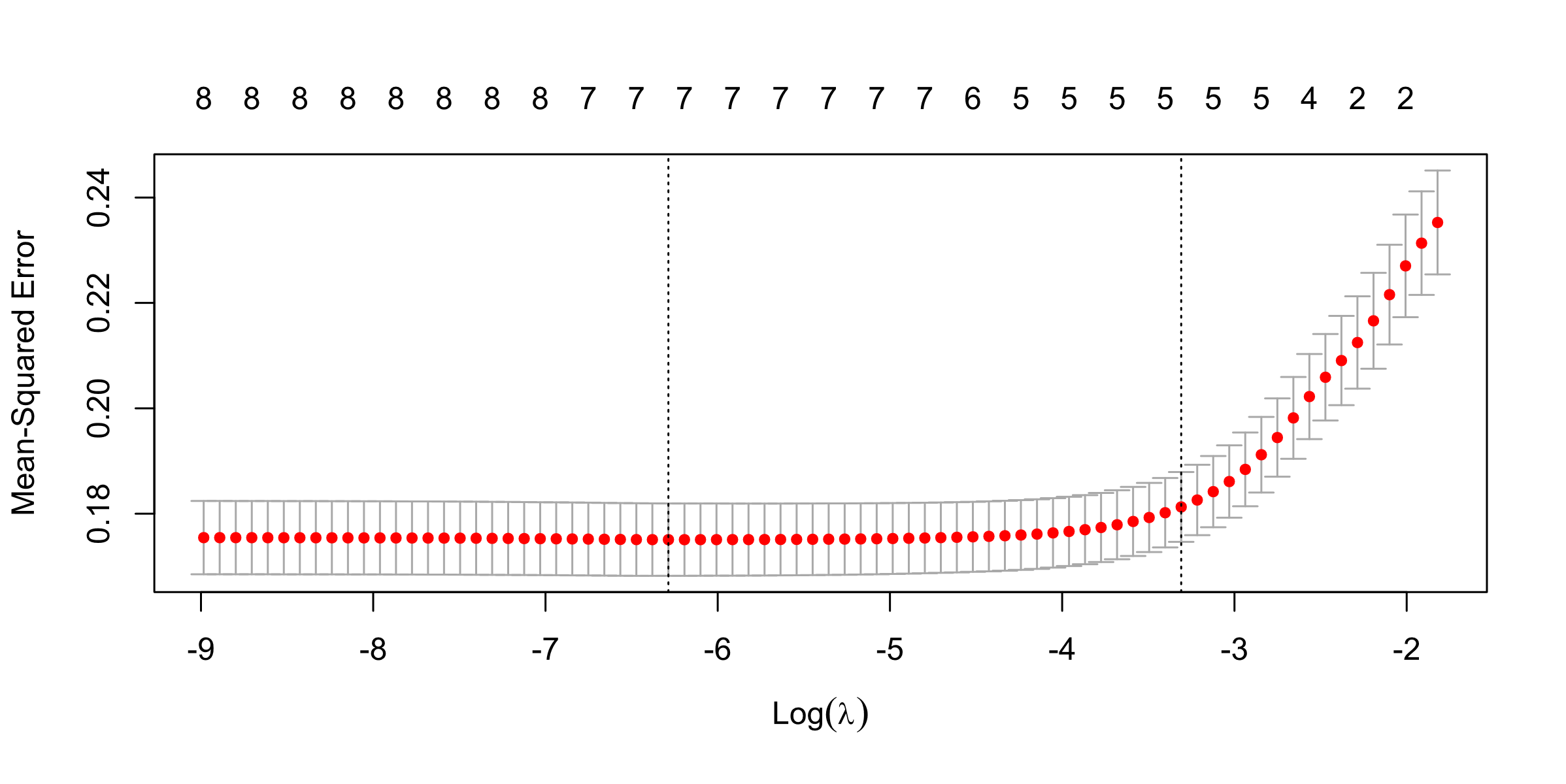


Best Lambda: 0.001861785

s1

(Intercept) 2.345430406

GenderFemale .

BMI 0.034272298

FS3T -0.217736589

CI -0.102967016

Age -0.001115098

LVEDVi -0.001933936

Hct -0.186135320

GenderFemale:BMI 0.008324355

R² = 0.269801
